# Chromatin Targeting of HIPK2 Leads to Acetylation-Dependent Chromatin Decondensation

**DOI:** 10.3389/fcell.2020.00852

**Published:** 2020-09-01

**Authors:** Jana Haas, Daniel Bloesel, Susanne Bacher, Michael Kracht, M. Lienhard Schmitz

**Affiliations:** ^1^Institute of Biochemistry, Justus-Liebig-University, Giessen, Germany; ^2^Member of the German Center for Lung Research, Giessen, Germany; ^3^Rudolf-Buchheim-Institute of Pharmacology, Justus-Liebig-University, Giessen, Germany

**Keywords:** HIPK2, chromatin decondensation, acetylation, estrogen receptor, chromatin

## Abstract

The protein kinase homeodomain-interacting protein kinase 2 (HIPK2) plays an important role in development and in the response to external cues. The kinase associates with an exceptionally large number of different transcription factors and chromatin regulatory proteins to direct distinct gene expression programs. In order to investigate the function of HIPK2 for chromatin compaction, HIPK2 was fused to the DNA-binding domains of Gal4 or LacI, thus allowing its specific targeting to binding sites for these transcription factors that were integrated in specific chromosome loci. Tethering of HIPK2 resulted in strong decompaction of euchromatic and heterochromatic areas. HIPK2-mediated heterochromatin decondensation started already 4 h after its chromatin association and required the functionality of its SUMO-interacting motif. This process was paralleled by disappearance of the repressive H3K27me3 chromatin mark, recruitment of the acetyltransferases CBP and p300 and increased histone acetylation at H3K18 and H4K5. HIPK2-mediated chromatin decompaction was strongly inhibited in the presence of a CBP/p300 inhibitor and completely blocked by the BET inhibitor JQ1, consistent with a causative role of acetylations for this process. Chromatin tethering of HIPK2 had only a minor effect on basal transcription, while it strongly boosted estrogen-triggered gene expression by acting as a transcriptional cofactor.

## Introduction

Mammalian homeodomain-interacting protein kinases (HIPKs) 1–4 are serine/threonine kinases and belong to the group of CMGC kinases (Manning et al., 2002). The HIPKs are phylogenetically most closely related to the subfamily of dual-specificity tyrosine phosphorylation regulated kinases (DYRKs), a kinase family with a role in cell cycle control, chromatin remodeling, transcriptional control and alternative mRNA splicing (Soppa and Becker, 2015). The kinases HIPK1-3 share an N-terminal kinase domain that is followed by a protein-protein interaction domain and a speckle retention sequence (SRS). The SRS contains a SUMO-interacting motif (SIM) allowing non-covalent binding of the SUMO isoforms SUMO-1-3 (De La Vega et al., 2011; Sung et al., 2011). Functional analysis of the HIPK2 SIM showed its relevance for protein/protein interactions, consistent with the general function of SUMO/SIM interactions as a molecular glue that serves to facilitate the dynamic regulation of protein assemblies (Hecker et al., 2006; De La Vega et al., 2011). Another characteristic feature of HIP kinases is the cis-autophosphorylation of a critical tyrosine residue in the activation loop (Saul et al., 2013; Siepi et al., 2013; van der Laden et al., 2015), a mechanism ensuring that all synthesized HIPKs are already -at least partially- active even in the absence of additional signals. Control of HIP kinases involves dynamic changes in their intracellular localization, and also regulation of their protein abundance by multiple levels including regulated synthesis and decay (D’Orazi et al., 2012; Ohnheiser et al., 2015; Wong et al., 2020). The best studied member of the HIPK family is HIPK2, a kinase that is involved in many different biological processes including the maintenance of basal cardiac functions, cell abscission, type I interferon-mediated antiviral immunity and neuronal survival (Shang et al., 2018; Guo et al., 2019; Pisciottani et al., 2019). In addition, HIPK2 contributes to developmental and differentiation processes to control adipogenesis (Sjölund et al., 2014), the development of various neuronal subtypes, angiogenesis, vasculogenesis, and hematopoiesis in different contexts (Blaquiere and Verheyen, 2017). All these different functions are mediated by the contribution of HIPK2 to a bewildering number of different signaling pathways including Wnt/Wingless, TGFβ, Notch and p53 (D’Orazi et al., 2002; Hofmann et al., 2002; Harada et al., 2003; Kanei-Ishii et al., 2004; Lee et al., 2009).

HIPK2 is enriched in small speckles in the cytosol and the nucleus and accordingly it exerts its functions in both compartments (Ritter and Schmitz, 2019). HIPK2-mediated protein/protein interactions have been found for cytosolic proteins, while most substrates are nuclear. The largest group of HIPK2 interactors are transcription factors including p53, Nkx1.2, Pax6, CREB1, ATF1/2, c-Jun, FOXM1, MEF2C, NRF2, and c-Myb (Kim et al., 1998, 2006; D’Orazi et al., 2002; Hofmann et al., 2002, 2003; Hailemariam et al., 2010; De La Vega et al., 2013; Hashimoto and Tsuji, 2017; Torrente et al., 2017; Liu et al., 2019). In addition, HIPK2 can regulate gene expression programs by its ability to interact with writers, readers and erasers of the chromatin modifications and with chromatin regulatory proteins. HIPK2-associated writers of chromatin modifications include the acetyltransferases CBP and p300 (Hofmann et al., 2002; Aikawa et al., 2006). While HIPK2 phosphorylates both acetyltransferases at multiple residues to enhance their coactivator activity, *vice versa* also CBP/p300 can acetylate HIPK2 to control its function (De La Vega et al., 2012; Choi et al., 2017). HIPK2-associated chromatin readers include proteins binding to methylated DNA such as methyl-CpG-binding protein 2 (MeCP2) (Bracaglia et al., 2009) or ZBTB4 (Yamada et al., 2009). Another HIPK2-associated chromatin reader protein is the polycomb protein Pc2/CBX4, a member of the multiprotein complex polycomb repressive complex 1 (PRC1) (Saurin et al., 1998; Roscic et al., 2006). Pc2/CBX4 has a chromodomain interacting with the repressive H3K27me3 histone mark and mediates transcriptional repression together with the PRC2 complex (Kaustov et al., 2011; Luis et al., 2011). The group of HIPK2-associated erasors includes the histone deacetylases HDAC3, HDAC4, and HDAC7 (De La Vega et al., 2013; Shang et al., 2013), but also the NAD + -dependent deacetylase SIRT1 (Conrad et al., 2016). While the association of HIPK2 with transcription factors typically leads to an increased gene expression, also gene repressive functions of HIPK2 have been described. For example, HIPK2 also engages in complexes with NK-3 and the corepressors Groucho and mSin3A to repress transcription (Choi et al., 1999, 2005). Similarly, HIPK2 together with c-Ski represses BMP-mediated transcription, while HIPK2 in complex with Brn3a mediates transcriptional repression of sensory neuron survival (Harada et al., 2003; Wiggins et al., 2004). In addition, HIPK2 can function as a corepressor to inhibit MEF2-dependent gene expression in undifferentiated myoblasts (De La Vega et al., 2013).

Dependent on the cellular context and signaling pathway, HIPK2 has been found as a facilitator or repressor of gene expression, but its function on chromatin compaction has not yet been directly tested. To address this question, we fused HIPK2 to the specific DNA-binding proteins LacI and Gal4 in order to allow specific tethering to well-defined heterochromatic or euchromatic loci in cells engineered to contain multimerized binding sites for these transcription factors. This experimental strategy has been successfully employed to study the dynamic chromatin-regulatory functions of proteins (Ahanger et al., 2014; Weth et al., 2014), an experimental approach that is specifically suitable for proteins lacking DNA-binding domains that are difficult to analyze by chromatin immunoprecipitation (ChIP). These tetherting approaches revealed a HIPK2-mediated increase in histone acetylations and a concomitant chromatin decondensation. While HIPK2 only weakly triggers transcription, it serves as a powerful coactivator for estrogen-induced gene expression.

## Materials and Methods

### Cell Culture and Transfection

U2OS F42B8 cells (Jegou et al., 2009), MCF7, 293T-Gal4, and 293T cells were cultivated in Dulbecco’s modified Eagle medium (DMEM, high glucose, GlutaMax, pyruvate, phenol red; Life Technologies) supplemented with 10% (v/v) fetal calf serum, 100U/ml penicillin, and 100μg/ml streptomycin. CHO RREB1 cells (Robinett et al., 1996) were grown in F12 medium containing the same supplements. Cells were grown in an incubator at 37°C in a humidified atmosphere containing 5% CO_2_. For estrogen stimulation experiments, cells were grown in DMEM without phenol red containing 10% (v/v) charcoal-stripped FBS, 100U/ml penicillin, 4 mM GlutaMax and 1 mM sodium pyruvate (CSS-DMEM). MCF7 cells were transfected using Lipofectamine 3000 (Invitrogen) according to the manufacturer’s recommendations. All the other cell lines were transfected using linear polyethylenimine (2.5 μg per μg of DNA) in serum- and antibiotic-free DMEM and incubated at room temperature for 30 min. Growth medium of cells was changed to antibiotic-free DMEM after one wash with warm phosphate-buffered saline (PBS). The transfection mix was then added to the cells in a dropwise manner and was gently swirled to mix. After 4 h the transfection medium was removed and cells were further grown until harvest.

### Immunofluorescence and Microscopy

Cells were grown on coverslips in 12-well plates and transfected and/or treated as described in the figure legends. Cells were washed twice with PBS and then fixed for 1 min with ice-cold methanol/acetone (1:1). After air drying, cells were rehydrated for 10 min and blocking solution [PBS containing 10% (v/v) BSA] was added for 1 h at room temperature. Coverslips were subsequently incubated with the indicated primary antibodies, diluted in PBS [containing 1% (v/v) BSA and 0.1% (v/v) Triton X-100] for 90 min at room temperature or at 4°C overnight. After washing three times with PBS, cells were incubated with the appropriate secondary Cy3-conjugated antibodies (Jackson ImmunoResearch) diluted 1:3000 in PBS containing 1% (w/v) BSA for 90 min in the dark. After incubation, cells were washed three times in PBS and the nuclear DNA was stained with Hoechst 33324 (Invitrogen). After washing with PBS, the samples were mounted with Kaiserś Glycerol Gelatine or with Mowiol mounting medium, sealed and stored in the dark at 4°C. Immunofluorescence staining of cells with histone-specific antibodies was done using the following buffers: Fixing buffer: 4% (w/v) paraformaldehyde in PBS (pH 6,9). Permeabilization buffer: PBS containing 0,15% (v/v) Triton X-100. Blocking solution: PBS containing 5% (v/v) BSA or goat serum, 0.05% (v/v) Tween-20. Antibody solution: similar to blocking solution but with 2% (w/v) BSA. Healthy interphase cells were analyzed using an Eclipse TE2000-E microscope (Nikon) equipped with a X-Cite Series 120 light source (EXFO), a T-RCP controller (Nikon) and a ORCA SPARK camera (Hamamatsu). To ensure inter-experimental comparability, all experiments were done with the identical microscopy settings using the Plan Apo 100x Oil Ph3 DM lens. Quantification of GFP spots indicating lacO/LacI arrays was done using the program NIS elements AR 3.0 to calculate the areas using the *Autodetect* function. Images were further processed using ImageJ, all experiments were statistically evaluated.

### Chromatin Immunoprecipitation

293T-Gal4 cells (2 × 10^7^) were transfected as displayed in the figures. After 2 days, cells were treated with formaldehyde (1% (v/v) final concentration) for 10 min, followed by addition of glycine (0,1 M final concentration) for 5 min. 10 ml of cold PBS containing 1 mM freshly added phenylmethylsulfonylfluoride (PMSF) was added to the cells which were then transferred to a 50 ml Falcon tube. Cells were washed two more times with PBS, pelleted and resuspended in 1 ml lysis buffer [1% (w/v) SDS, 10 mM EDTA, 50 mM Tris/HCl pH 8.1, 0.5 mM PMSF, and a protease inhibitor cocktail (Roche)]. After 10 min incubation on ice, 1 ml of the lysate was transferred to a COVARIS sonicator to shear the genomic DNA. Sonicated lysates were centrifuged for 15 min at 13200 rpm at 4°C and supernatants transferred to new reaction tubes. Aliquots representing 25 μg of chromatin were diluted 1:10 with dilution buffer [0.01% (w/v) SDS, 1.1% (v/v) Triton X-100, 1.2 mM EDTA, 167 mM NaCl, 16,7 mM Tris/HCl pH 8.1] and subjected to 4 h of preclearing with an agarose A/G-bead-mixture and 2 μg rabbit IgG antibody at 4°C with end-over-end tumbling. Supernatants were incubated with the indicated antibodies or rabbit IgG antibody. Immunoprecipitation was carried out over night with end-over-end tumbling at 4°C. A mixture of agarose A/G-beads was added for 4 h, afterward immunoprecipitated complexes were successively washed for 5 min at 4°C with end-over-end tumbling with low-salt buffer [0.1% (w/v) SDS, 1% (v/v) Triton X-100, 2 mM EDTA, 150 mM NaCl, 20 mM Tris/HCl pH 8.1], high-salt buffer [0.1% (w/v) SDS, 1% (v/v) Triton X-100, 2 mM EDTA, 500 mM NaCl, 20 mM Tris/HCl pH 8.1], LiCl-buffer [250 mM LiCl, 1% (v/v) NP40, 1% (w/v) deoxycholate, 1 mM EDTA, 10 mM Tris/HCl pH 8.1], and twice with TE-buffer (10 mM Tris/HCl pH 8.1, 1 mM EDTA). Reverse-crosslinking took place in TE-buffer with addition of RNase A for 30 min and Proteinase K for 2 h at 37°C followed by incubation at 65°C over night. Free DNA was purified using NucleoSpin Gel and PCR Clean Up Kit with buffer NTB (Macherey-Nagel) and was eluted in 50 μl water. Subsequently qPCR experiments were performed, the reaction mixture contained 2 μl of ChIP or input DNA (diluted 1:10), signals were calculated as % input. Results from modification-specific histone antibodies were also normalized to ChIP experiments using pan-specific antibodies recognizing the histones.

### FAIRE Experiments

293T-Gal4 cells were transfected as indicated in the figure legend. After 2 days, cells were crosslinked by adding formaldehyde [1% (v/v) final concentration] directly to the cell culture medium and incubated 10 min at room temperature. Further processing of the samples was essentially done as described (Rodriguez-Gil et al., 2018). Briefly, glycine (0.125 M final concentration) was added, followed by incubation for 5 min at room temperature to quench crosslinks. 10 ml of cold PBS containing 1 mM freshly added PMSF was added to the cells which were then transferred to a 50 ml Falcon tube. Cells were washed two more times with PBS, pelleted and resuspended in 1 ml lysis buffer [1% (w/v) SDS, 10 mM EDTA, 50 mM Tris HCl pH 8.1, 1 mM PMSF, 10 mM NaF, 0.5 mM sodium orthovanadate, 10 μg/ml leupeptin, 10 μg/ml aprotinin]. After 20 min of incubation on ice, samples were sonified using a COVARIS sonicator to shear the DNA (final DNA fragment size ∼300 bp). One aliquot of each sample was treated with 5 μl of proteinase K and incubated for 4 h at 37°C, followed by an incubation for 6 h at 65°C to reverse the crosslink, or left untreated. All samples were then extracted by phenol/chloroform and the free DNA contained in the upper phase was quantified by qPCR. The fraction of free DNA was determined by dividing the amount of DNA obtained in the untreated sample by the amount obtained in the proteinase K-treated sample.

### Plasmids and Reagents

This information is listed in Supplementary Table 1.

### Western Blotting and Luciferase Experiments

NP-40 cell extracts were prepared and separated by denaturing SDS-PAGE as described (Saul et al., 2019). Proteins from SDS gels were transferred to polyvinylidene fluoride (PVDF) membranes using a semi-dry blotting apparatus (Bio-Rad) and 1 x transfer buffer [50 mM Tris; 40 mM glycine; 20% (v/v) methanol; 0.04% (w/v) SDS]. The electrophoretic transfer was performed at 24 V for 1–3 h, depending on the size of the proteins. After completion of protein transfer, the membranes were incubated in blocking buffer {5% (w/v) skimmed milk powder in TBS-T [25 mM Tris (pH 7.4); 137 mM NaCl; 5 mM KCl; 0.7 mM CaCl_2_; 0.1 mM MgCl_2_; 0.1% (v/v) Tween 20]}at room temperature for 1 h. The membranes were then incubated with the primary antibody solution at 4°C over night. Membranes were washed 5 × in TBS-T, followed by incubation with the secondary antibody solution for 1.5–2 h. The membrane was then washed again 5 times in TBS-T and proteins were detected on a ChemiDoc Touch imaging system (Bio-Rad) using enhanced chemiluminescence (ECL). Quantitative analysis of Western blot data was done in ImageLab 6.0.1 (Bio-Rad). Luciferase assays using the 293T-Gal4 cells were performed by transfection of the plasmids encoding the various Gal4 fusion proteins together with 100 ng of the plasmid encoding Renilla luciferase under the control of a constitutive SV40 promoter. After 2 days, cells were lysed in NP-40 lysis buffer as described (Saul et al., 2019) and extracts were either used for Western blotting or for determination of luciferase activities using the Dual Luciferase Assay System (Promega). Firefly luciferase activity was measured by mixing 2 μl of the extract with 10 μl of LAR II reagent, followed by determination of light emission using a Lumat LB9507 (Berthold Technologies). Renilla activity was measured in the same tube by adding 10 μl of Stop&Glo reagent, followed by determination of emitted light. Luciferase assays measuring ER-inducible gene expression were performed by transfection of cells with plasmids for lacO-(ERE)3-luciferase and SV40 Renilla luciferase together with plasmids encoding GFP-LacI-HIPK2, followed by further cultivation of cells in CSS-DMEM for 1 day. Then cells were treated either with DMSO as a vehicle control or with 17β-Estradiol (10 nM) for 8 h, followed by cell lysis in passive lysis buffer and determination of Renilla and Firefly luciferase activities. In all luciferase experiments, Firefly luciferase activities were normalized to the Renilla luciferase activities.

## Results

### Ectopically Tethered HIPK2 Mediates Chromatin Decondensation

**FIGURE 1:**
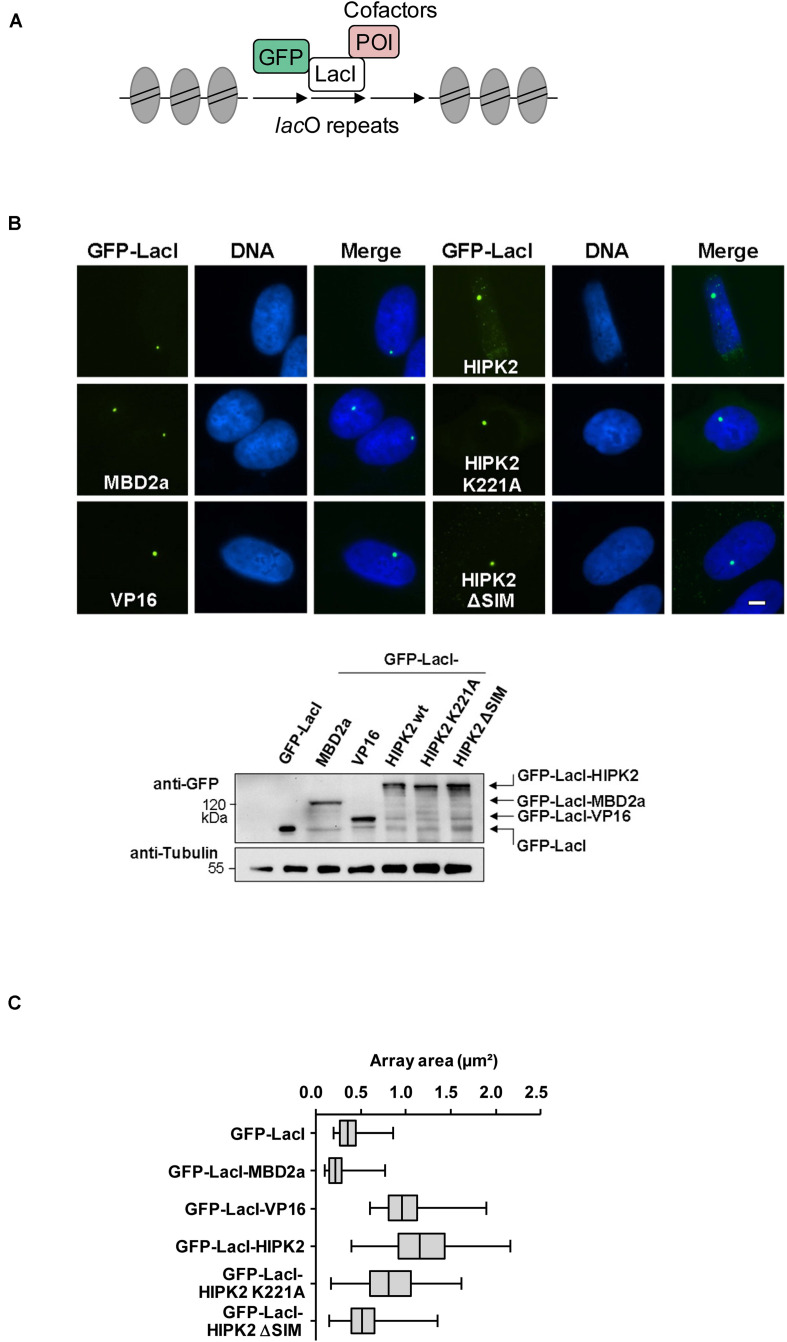
Cell biological analysis of HIPK2-mediated heterochromatin decompaction. **(A)** Schematic display of the lacO/LacI system, POI = protein of interest. **(B)** Upper: U2OS F42B8 cells were grown on cover slips and transfected with the indicated GFP-LacI expression plasmids. One day later cells were fixed and DNA was stained using Hoechst 33342. Representative results are displayed, the size bar represents 5 μm. Lower: 293T cells grown on a 6-cm dish were transfected as shown and cell lysates were analyzed for correct expression of the proteins using GFP antibodies. Note that HIPK2 K221A runs slightly faster due to lacking autophosphorylation. The position of a molecular weight marker is shown, Tubulin was detected to ensure comparable protein loading. **(C)** NIS-Elements AR software was used for the area quantification of 100 GFP spots per construct. The box plot displays the data between the first and third quartiles and the median is displayed. Whiskers extend to the smallest and largest data values.

The endogenous HIPK2 protein is tethered to the chromatin by association with numerous transcription factors. To investigate the functional consequences of this process in an experimental surrogate system, we used the lacO-LacI tethering assay. This system consists of two components, as schematically shown in Figure 1A. The first component consists of a fusion protein expressing the prokaryotic DNA-binding domain of the lac repressor (LacI) in fusion with GFP and a protein of interest. The second component is a cell line engineered to contain repetitive binding sites for LacI (lacO) integrated either at hetero- or euchromatic regions. Expression of the fusion protein leads to tethering at the lacO repeats and the GFP allows to monitor localization of the fusion protein and also potential changes in the condensation state of the lacO array (Robinett et al., 1996). As the prokaryotic LacI protein does not occur in mammalian cells, the effects of tethering are direct and not blurred by indirect effects, as it could occur upon fusion of HIPK2 to a mammalian transcription factor that could induce gene expression programs.

**FIGURE 2:**
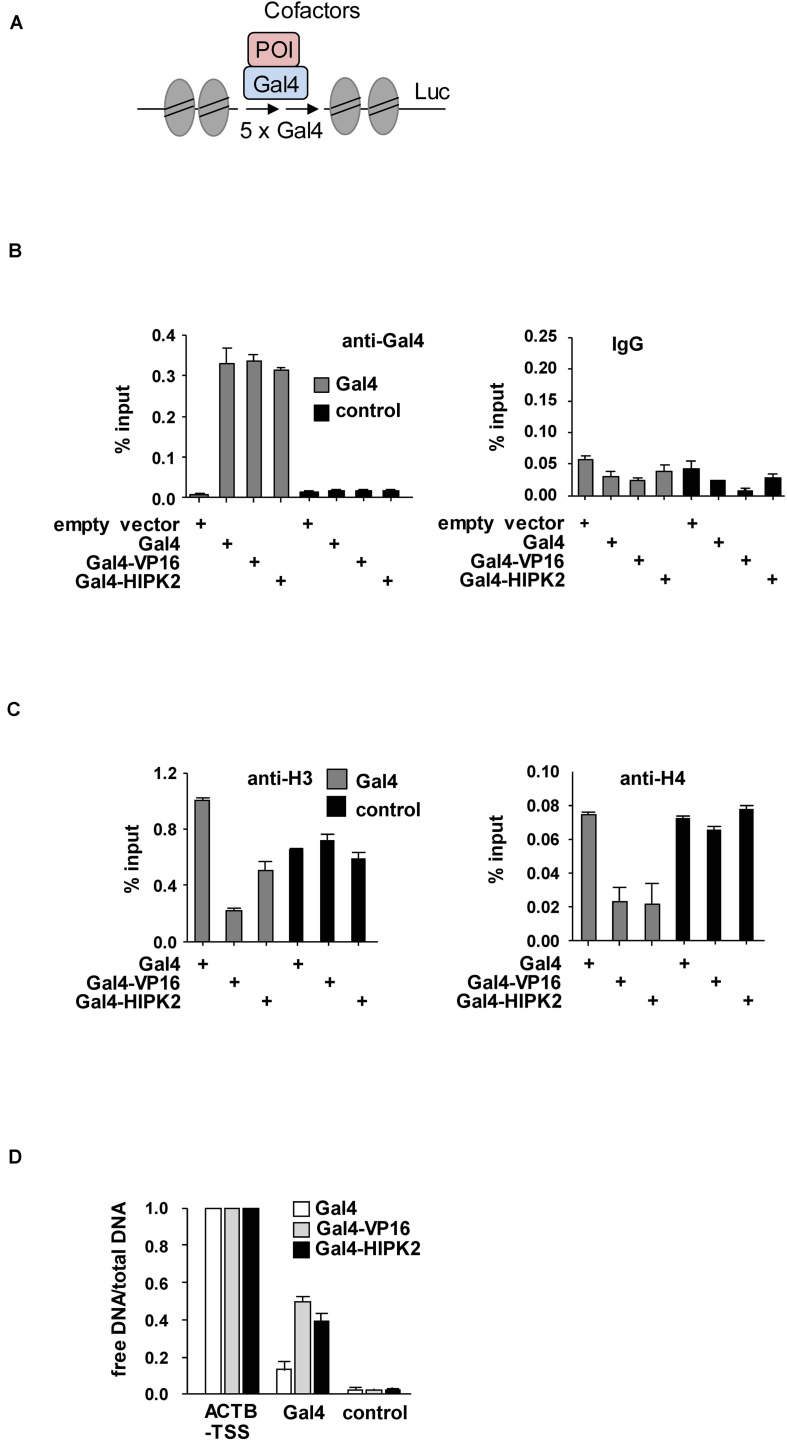
Biochemical analysis of HIPK2-mediated heterochromatin decompaction. **(A)** Schematic display of the stably integrated Gal4 sites controlling expression of the luciferase (Luc) gene. **(B)** 293T-Gal4 cells were transfected as shown. After 2 days cells were fixed and subjected to ChIP assays using Gal4 antibodies or matched control antibodies. The qPCR reaction detected either the chromatin region encompassing the Gal4 binding sites or a gene desert in chromosome 2 (control). The error bars show standard deviations from three independent experiments. **(C)** The experiment was performed as in **(B)** with the exception that histone H3 or H4 were used for ChIP. **(D)** 293T-Gal4 cells were transfected to express Gal4, Gal4-VP16, or Gal4-HIPK2 as shown. The ratio between free vs. total DNA (i.e., the relative recovery ratio) was determined for the promoter of the *ACTB* gene as a positive control, a gene desert as a negative control, and a region adjacent to the Gal4 sites as shown. The error bars show standard deviations from three independent experiments.

To investigate a potential effect of HIPK2 on chromatin compaction, we expressed moderate amounts of a GFP-LacI-HIPK2 fusion protein in U2OS F42B8 cells containing 256 lacO repeats integrated into a single heterochromatic region at the pericentromere of chromosome 2 (Jegou et al., 2009). Tethering of HIPK2 to the lacO repeats resulted in a decondensation of heterochromatin, as revealed by expansion of the single GFP spot (Figure 1B). The expansion of the lacO array induced by HIPK2 was as pronounced as chromatin decondensation caused by tethering of the transactivation domain of VP16 from Herpes simplex virus, which is well known for its ability to decondensate chromatin (Weth et al., 2014). In agreement with published literature, recruitment of the methyl-CpG-binding domain protein 2 (MBD2) to the lacO arrays caused chromatin compaction (Günther et al., 2013), showing that the employed U2OS F42B8 cell system allows monitoring of the full dynamic range of chromatin (de)compaction. It was then interesting to test a possible contribution of HIPK2 kinase function and non-covalent SUMO interaction on its ability to mediate chromatin decompaction. While a HIPK2 mutant unable to bind to SUMO (HIPK2 ΔSIM) had largely lost its ability for chromatin decondensation, a kinase inactive HIPK2 mutant (HIPK2 K221A) still led to chromatin decompaction, as revealed by lacO-LacI tethering assays and their quantitative evaluation (Figures 1B,C). To test the ability of HIPK2 for chromatin opening by an independent experimental approach, we employed a 293T-Gal4 cell line with a single copy of a Firefly luciferase gene under the control of 5 binding sites for the yeast transcription factor Gal4 stably integrated into chromosome 20 (Stielow et al., 2008). Also this system allows chromatin recruitment of proteins, but in this case tethering requires fusion to the DNA-binding domain of the yeast transcription factor Gal4, as schematically depicted in Figure 2A. In order to test the functionality of the system we expressed Gal4 alone or in fusion with VP16 or HIPK2 and tested appropriate targeting to the Gal4 binding sites in 293T-Gal4 cells using ChIP experiments. ChIP assays using anti-Gal4 antibodies revealed appropriate and specific binding of all Gal4 proteins to the chromosomal region containing the Gal4 binding sites (Figure 2B). To investigate the effects of Gal4-HIPK2 on chromatin condensation, this fusion protein was expressed in 293T-Gal4 cells, followed by ChIP experiments detecting either histone H3 or H4 in the regions encompassing the Gal4 binding sites. These experiments showed a clear reduction in H3/H4 occupancy after expression of Gal4-HIPK2 (Figure 2C), indirectly showing that tethering of this kinase to a specific locus leads to reduced nucleosome density in the vicinity. The ability of HIPK2 for chromatin decompaction was comparable to that of VP16 also in these experiments. These findings were further corroborated by FAIRE (Formaldehyde-Assisted Isolation of Regulatory Elements) assays, which determine chromatin accessibility (Giresi et al., 2007). Expression of Gal4-HIPK2 in 293T-Gal4 cells caused an increase in protein-depleted DNA in a chromosomal region adjacent to the Gal4 sites (Figure 2D), indicating that HIPK2 leads to reduced protein occupancy.

**FIGURE 3:**
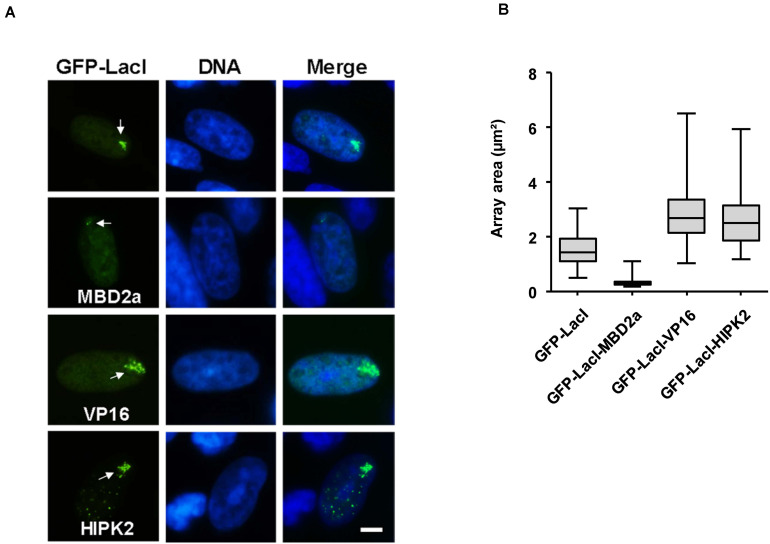
Cell biological analysis of HIPK2-mediated euchromatin decompaction. **(A)** CHO RREB1 cells were grown on cover slips and transfected with the indicated GFP-LacI expression plasmids. One day later cells were fixed and DNA was stained using Hoechst 33342. Representative results are shown, the bar represents 5 μm. As the lacO array can interact only with a limited number of LacI fusion proteins, the faintly visible multiple HIPK2 speckles outside from the lacO array likely represent GFP-LacI-HIPK2 molecules that are not attached to the DNA and thus form HIPK2 speckles. **(B)** The areas of 100 GFP spots per construct were quantified and are displayed as box plots.

Can HIPK2 also lead to the further expansion of euchromatic areas? In order to address this question, the GFP-LacI-HIPK2 fusion protein was expressed in CHO RREB1 cells harboring lacO repeats in a euchromatic region (Robinett et al., 1996). Also in this context, HIPK2 was as potent as VP16 in its ability to further expand the chromatin region, as revealed by immunofluorescence analysis (Figure 3A) and its quantitative evaluation (Figure 3B).

### Chromatin-Associated HIPK2 Leads to Decreased H3 Lysine 27 Trimethylation (H3K27me3)

**FIGURE 4:**
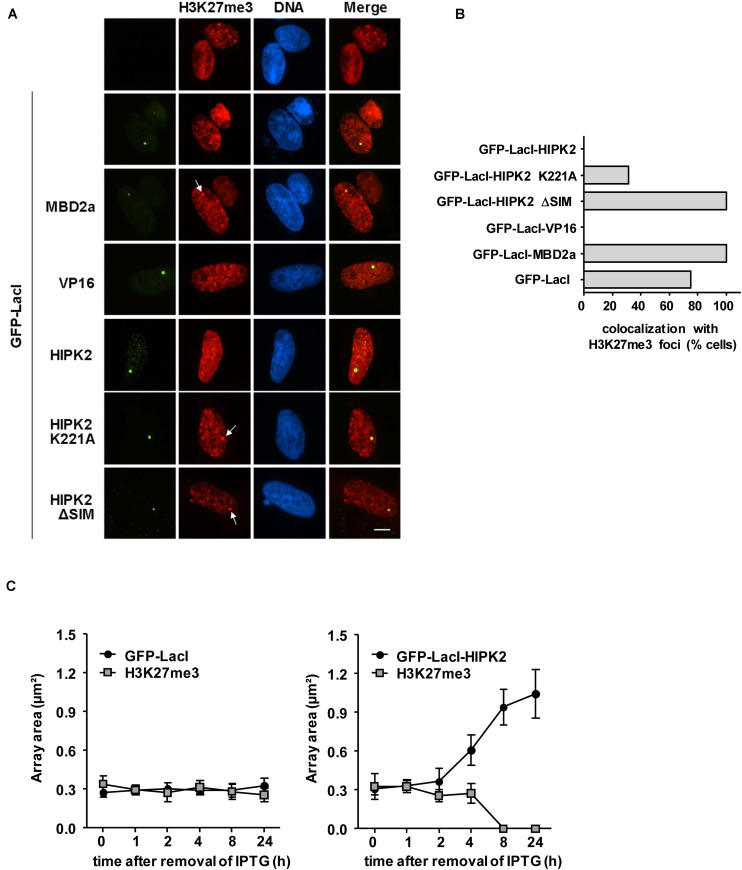
Anti-correlation between H3K27me3 and HIPK2. **(A)** U2OS F42B8 cells were transfected with the indicated plasmids. The next day, cells were fixed and stained for H3K27me3 using specific antibodies. Indirect immunofluorescence was used to analyze co-localization with lacO arrays emitting the GFP signal. Arrows point to H3K27me3 spots and the bar represents 5 μm. **(B)** The colocalization between H3K27me3 spots and 30 GFP spots/fusion protein was manually counted. **(C)** Plasmids encoding GFP-LacI or GFP-LacI-HIPK2 were used to transfect U2OS F42B8 cells. Directly after transfection IPTG (150 μM) was added for 16 h to prevent DNA-binding of LacI. After removal of IPTG, cells were further cultivated for the indicated periods and subsequently stained with antibodies detecting H3K27me3. The areas of 100 GFP spots per construct were quantified and their colocalization with H3K27me3 foci was evaluated, data are displayed as box plots.

It was then interesting to determine whether HIPK2-mediated decondensation is accompanied by a decrease in repressive chromatin marks such as H3K27me3 (Hansen et al., 2008). GFP-LacI in fusion with HIPK2 or appropriate controls such as VP16 or MBD2a were expressed in U2OS F42B8 cells, followed by immunostaining for H3K27me3. Indirect immunofluorescence showed the occurrence of H3K27me3 in many nuclear regions with some focal enrichment in the GFP spot containing MBD2a (Figure 4A). In contrast, focal enrichment of H3K27me3 was not seen in areas enriched in GFP-LacI fused to HIPK2 or VP16. Interestingly, lacO recruitment of HIPK2 ΔSIM or kinase inactive HIPK2 still allowed colocalization with H3K27me3 spots, as revealed by immunofluorescence (Figure 4A) and its quantitative analysis (Figure 4B). It was then important to determine the kinetics of HIPK2-mediated disappearance of H3K27me3 spots and chromatin decompaction. To address this question, U2OS F42B8 cells were transfected to express GFP-LacI-HIPK2 or the vector control, followed by addition of the allosteric regulator isopropyl β-D-1-thiogalactopyranoside (IPTG), which prevents binding of LacI to its cognate DNA binding site (Gilbert and Müller-Hill, 1966). DNA-binding of LacI was induced by removal of IPTG, followed by a kinetic analysis of chromatin decompaction and the occurrence of H3K27me3 spots at the GFP foci. Immunofluorescence analysis showed that chromatin decompaction already started 4 h after IPTG removal and was maximal after 24 h (Figure 4C and Supplementary Figure 1). This kinetics was paralleled by the disappearance of H3K27me3 spots, showing that the decompaction process starts already a few hours after DNA-binding.

### Chromatin-Association of HIPK2 Leads to the Induction of Local Histone Acetylation

**FIGURE 5:**
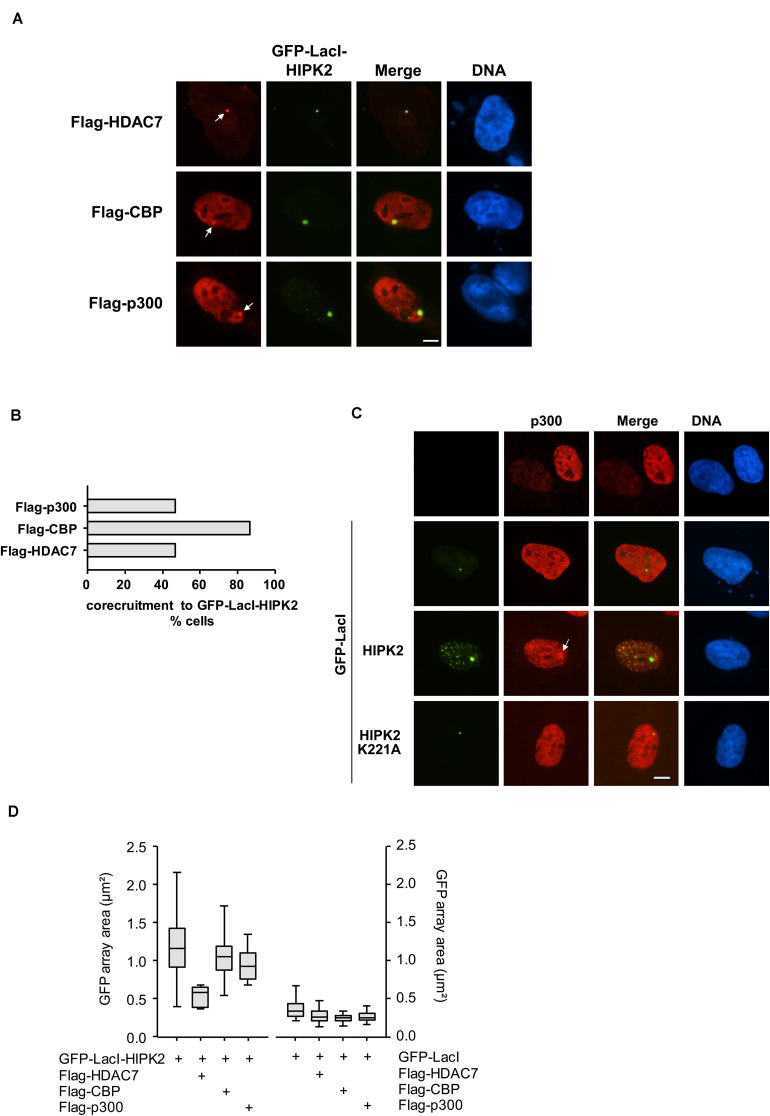
Co-localization of HIPK2 with (de)acetylating proteins. **(A)** U2OS F42B8 cells were transfected to express moderate amounts of GFP-LacI-HIPK2 together with the indicated epitope-tagged proteins. After 1 day, cells were fixed and analyzed by immunofluorescence for the intracellular localization of the labeled proteins and the GFP signal. The arrows point to lacO arrays, representative experiments are shown. **(B)** Quantitative analysis of the experiment displayed in **(A)**, 30 GFP spots per construct were analyzed. **(C)** GFP-LacI-HIPK2 or its kinase-inactive derivative were expressed in U2OS F42B8 cells, followed by immunofluorescence staining for the endogenous p300 protein. The arrow points to an area containing the lacO arrays and a focal enrichment of p300, a representative experiment is shown. Bars in all graphs represent 5 μm. **(D)** Cells were transfected with GFP-LacI-HIPK2 or the GFP-LacI control together with the indicated proteins as described for **(A)**. The areas of 100 GFP spots per construct were quantified, the data are presented as box plots.

**FIGURE 6:**
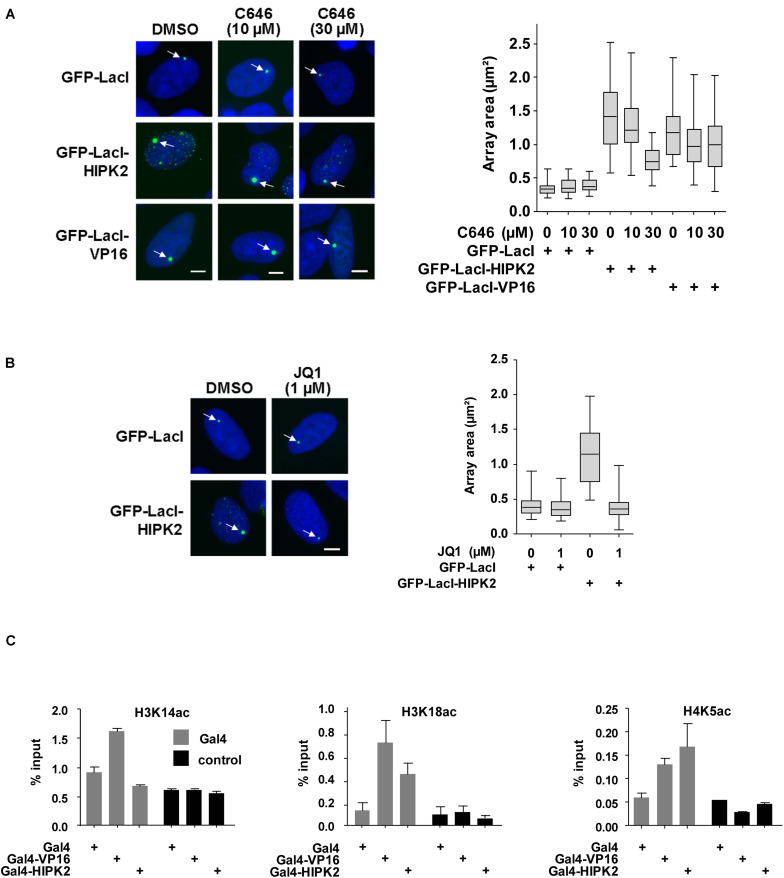
Histone acetylation contributes to HIPK2-mediated chromatin decompaction. **(A)** Left: U2OS F42B cells were transfected with the indicated GFP-LacI fusion proteins and incubated with IPTG for 16 h. After removal of IPTG cells were treated with the indicated amounts of C646 or solvent control for 1 day. Cells were fixed and analyzed by fluorescence microscopy, arrows point to the region containing the lacO repeats. Right: The areas of 50 GFP spots per construct were quantified, the data are presented as box plots. **(B)** The experiment was done as in **(A)** with the exception that cells were treated with JQ1 after removal of the IPTG. **(C)** 293T-Gal4 cells were transfected as shown. After 2 days cells were fixed and subjected to ChIP assays using either antibodies recognizing the acetylated histone proteins or the unmodified histones. The qPCR reaction detected either the chromatin region encompassing the Gal4 binding sites or a gene desert in chromosome 2 (control). Data are displayed as % input after normalization to H3 and H4, respectively. The error bars show standard deviations from three independent experiments.

Increased histone acetylation is frequently contributing to the conversion of heterochromatin to euchromatin (Hebbes et al., 1988). HIPK2 is known to functionally interact with acetyltransferases CBP and p300 and also with histone deacetylases including HDAC3, HDAC4, and HDAC7 (De La Vega et al., 2012, 2013; Shang et al., 2013), raising the possibility that it may indirectly affect histone acetylation. To investigate whether lacO-tethered HIPK2 has the ability to recruit (de)acetylases, U2OS F42B8 cells were transfected to express GFP-LacI-HIPK2 along with epitope-tagged versions of CBP, p300, HDAC3, HDAC4, or HDAC7. Both acetyltransferases as well as HDAC7 were enriched at GFP-LacI-HIPK2 foci, as revealed by immunofluorescence (Figure 5A and Supplementary Figure 2) and its quantitative analysis (Figure 5B). Also the endogenous p300 protein showed a largely nuclear localization with a zone of enrichment at the GFP-LacI-HIPK2-containing lacO arrays, which was not occurring in cells expressing a kinase inactive HIPK2 variant (Figure 5C). Coexpression of HDAC7 significantly limited the ability of HIPK2 for chromatin decompaction, as revealed by the reduced size of the GFP spot, suggesting a contribution of acetylation for HIPK2-mediated regulation of chromatin compaction. In contrast, HIPK2-mediated opening of the heterochromatic array was not further enhanced by coexpression of CBP or p300 (Figure 5D). These results suggest that the endogenous CBP/p300 proteins are already sufficient and not rate limiting in their ability to promote chromatin decondesation. To address this possibility experimentally, we tested the effect of the cell permeable CBP/p300 inhibitor C646 (Bowers et al., 2010) on HIPK2-mediated chromatin decompaction. The functionality of C646 was ensured in control experiments confirming its ability to interfere with histone acetylation (Supplementary Figure 3). Chromatin decompaction induced by expression of GFP-LacI-HIPK2 was significantly reduced by C646 in a dose-dependent manner (Figure 6A and Supplementary Figure 4), showing a contribution of the endogenous acetyltransferase activity for this process. Histone acetylation does not only alter the charge and sterical properties of chromatin, but also allows binding of bromodomain-containing proteins (Dhalluin et al., 1999). This interaction can be inhibited by the specific and reversible BET (Bromodomain and extraterminal domain) bromodomain inhibitor JQ1 (Filippakopoulos et al., 2010). To test a possible contribution of BET bromodomain proteins on HIPK2-mediated chromatin decompaction we performed a lacO/LacI tethering assay in the presence of JQ1, which resulted in a complete inhibition of HIPK2-mediated chromatin decompaction (Figure 6B and Supplementary Figure 5). As HIPK2-mediated chromatin decondensation relies -at least in part- on its ability to increase acetylation, it was then interesting to determine some of the affected acetylation sites. We thus investigated the possible GFP-LacI-HIPK2-induced acetylation using a number of different antibodies recognizing various histone acetylations (Supplementary Table 2). As the immunofluorescence analysis did either show only an enrichment for the VP16 control (Supplementary Figure 6) or no enrichment at the HIPK2 foci (Supplementary Table 1), the experimental strategy was changed. Gal4-HIPK2 or the Gal4-VP16 control were expressed in 293T-Gal4 cells, followed by ChIP experiments using various antibodies recognizing different histone acetylations. These experiments showed that elevated H3K4 acetylation was rather specific to VP16, while acetylation of H4K5 and H3K18 was also increased after tethering of HIPK2 (Figure 6C).

### HIPK2 Functions as a Transcription Co-activator

**FIGURE 7:**
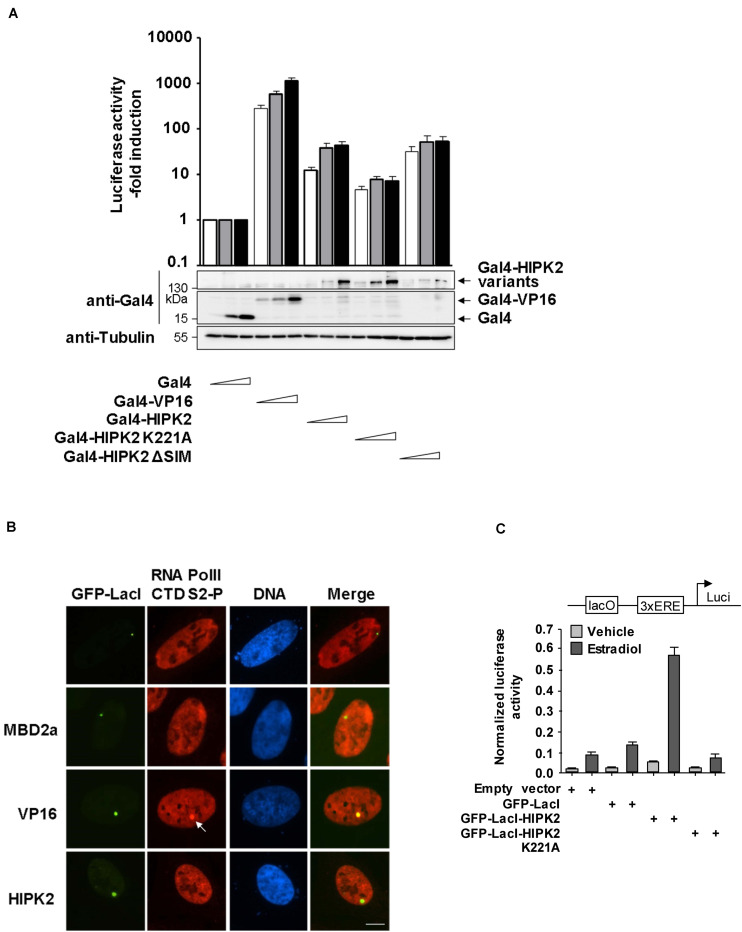
HIPK2 is a weak transactivator and rather functions as a cofactor for estrogen-induced gene expression. **(A)** 293T-Gal4 cells were transfected with increasing amounts of the indicated Gal4 plasmids along with a plasmid encoding a Renilla luciferase gene. After 2 days, cells were lysed and one aliquot was analyzed for luciferase activities. The relative activities were calculated after the normalization of the Firefly luciferase activities to the activities of the Renilla luciferase. Gene expression in cells expressing Gal4 was arbitrarily set as 1, error bars show standard deviations from three independent biological replicates. The other aliquot of cell lysates was tested by Western blotting for adequate expression of the transfected proteins. **(B)** U2OS F42B cells were transfected with the indicated GFP-LacI fusion proteins. The next day, cells were fixed and stained with the indicated phospho-specific antibodies, followed by immunofluorescence analysis. The arrow points to enriched CTD S2 phosphorylation at the region containing the lacO repeats. A representative experiment is shown, bar = 5 μm. **(C)** MCF7 cells were transfected with a luciferase reporter gene containing one lacO binding site and three EREs along with a Renilla luciferase construct and the indicated GFP-LacI fusion proteins. The next day, cells were treated for 8 h with 17β-Estradiol (10 nM), followed by cell lysis and determination of luciferase activity. Normalized values are shown, error bars show standard deviations obtained from two technical replicates.

To investigate whether HIPK2 directly activates transcription, 293T-Gal4 cells harboring stably integrated Gal4 sites controlling the expression of a luciferase gene were transfected to express increasing amounts of Gal4-HIPK2 and mutants thereof together with adequate controls. Expression of Gal4-HIPK2 increased gene expression only moderately, while transcription induced by Gal4-VP16 was almost 100 times higher (Figure 7A). The mild transcription induction by HIPK2 was dependent on its kinase activity, as the Gal4-HIPK2 K221A mutant was less active. To corroborate these findings by an independent experimental approach we used the lacO/LacI system and expressed GFP-LacI-HIPK2 or controls, followed by the indirect analysis of gene expression by staining the cells for phosphorylation of the RNA polymerase II C-terminal domain (CTD). Only cells expressing GFP-LacI-VP16 showed lacO areas enriched in CTD S5 phosphorylation, which is indicative for initiating polymerases (Supplementary Figure S7) or S2 phosphorylation (Figure 7B), which is used as a proxy for elongating RNA polymerase II (Eick and Geyer, 2013). These CTD phosphorylations were not enriched at GFP spots with tethered HIPK2, supporting the concept that HIPK2 only mildly affects transcription and rather leads to chromatin decompaction. While these data show that HIPK2 by itself has only a very low gene-activating capacity it was interesting to investigate whether it can support gene expression triggered by other transcription factors. As a coactivator function of HIPK2 for androgen receptors has already been described (Imberg-Kazdan et al., 2013; Bengtsen et al., 2017), we tested a possible contribution of HIPK2 to gene induction by a different hormone receptor. Toward this goal we created a plasmid where a lacO binding site allowing the tethering of GFP-LacI-HIPK2 was inserted upstream from three copies of an estrogen receptor response element (ERE) and a luciferase reporter gene. This plasmid was transfected in hormone-sensitive MCF-7 breast cancer cells in the presence or absence of GFP-LacI-HIPK2, followed by stimulation of gene expression upon addition of Estradiol. These experiments showed that estrogen receptor-induced gene expression was strongly triggered by HIPK2 tethering, while a kinase inactive version failed to support transcription (Figure 7C), thus demonstrating a kinase-dependent coactivator function of HIPK2.

## Discussion

### The Relevance of HIPK2 Kinase Function and SUMO Interaction for Chromatin Decondensation

Kinases of the HIPK family have the ability to regulate gene expression either negatively or positively. To investigate chromatin regulatory functions of HIPK2, the most extensively studied member of this kinase family, we used various tethering approaches. These revealed that a local increase in HIPK2 proteins leads to decondensation of surrounding chromatin areas, regardless whether they are heterochromatic or euchromatic. These results raise the possibility that HIPK2-mediated gene repression is rather mediated by its specific association with corepressors such as Groucho or mSin3A and probably also histone deacetylating enzymes in a cell type- and context-specific manner. The kinase function was not required for the chromatin decompaction, which could be either due to the potential association of endogenous wildtype kinases or alternatively indicate a kinase-independent function of HIPK2. We did not observe a HIPK2-mediated increase in histone phosphorylations (H2A.XS139, H3S10ph and H2BS6ph) using the lacO/LacI tethering system, a result that of course does not exclude further phoshorylation-dependent processes.

Interestingly, the SUMO-binding activity of HIPK2 was important for chromatin opening, consistent with the concept that extensive SUMO/SIM interactions are a molecular glue that contributes to many aspects of chromatin regulation (Cubenas-Potts and Matunis, 2013). We previously found that the HIPK2 ΔSIM mutant has lost its ability to interact with its interaction partners Pc2/CBX4 or PML in intact cells, supporting the concept that SUMO/SIM interactions stabilize protein/protein interaction networks (De La Vega et al., 2011). This may also affect the ability of HIPK2 to interact with chromatin proteins and transcription factors, which represent the largest class of SUMO targets (Eifler and Vertegaal, 2015). Genome-wide studies showed that SUMO is widely distributed over the genome with strong association at active promoters, while active SUMOylation occurs most prominently at promoters controlling the expression of genes encoding histones and ribosomal proteins (Liu et al., 2012; Neyret-Kahn et al., 2013). The SUMO chromatin landscape is dynamically changed in response to external cues including oncogene-induced senescence (Neyret-Kahn et al., 2013), heat shock (Niskanen et al., 2015; Seifert et al., 2015) as well as inflammatory and anti-viral gene expression (Decque et al., 2016). SUMOylation also enforces distinct chromatin states to safeguard somatic and pluripotent cell identities (Cossec et al., 2018) and it will be interesting to identify the SUMO-modified proteins mediating HIPK2-dependent chromatin decompaction in the future.

### HIPK2-Mediated Chromatin Opening Involves Increased Histone Acetylation

We assume that the association of HIPK2 with CBP and p300 will not be affected by SUMO/SIM interactions, as the SIM-containing C-terminus of HIPK2 is not required for this interaction (Hofmann et al., 2002). Association between both HATs and HIPK2 typically occurs in a complex with transcription factors including C/EBPβ, RUNX1/PEBP2-β, AML1, p53 and Pax6, suggesting that this complex is preferentially formed at the chromatin (Hofmann et al., 2002; Aikawa et al., 2006; Wee et al., 2008; Steinmann et al., 2013). The association of HIPK2 with HATs increases their coactivator function, which can involve an elevated HAT activity, as shown by *in vitro* experiments for the p300 protein (Aikawa et al., 2006). In addition, HIPK2 can activate CBP by counteracting the repressive action of cell cycle regulatory domain 1 (Kovacs et al., 2015). Also other members of the HIPK family can associate with p300. It was shown that c-Myb associates with the coactivator androgen receptor-associated protein 55 (ARA55) to form a complex with HIPK1/p300, thus strongly enhancing c-Myb transcriptional activity (Bengtsen et al., 2017). The association of HIPK2 with HATs and HDAC7 was independent from the chromatin state, as we observed co-localization between these proteins also in CHO RREB1 cells harboring lacO repeats in an euchromatic region (Supplementary Figure S8).

We suggest that HIPK2-assisted recruitment of HATs lead to an increase of specific histone acetylations at various sites including H3K18, a modification that is enriched at transcription start sites (Wang et al., 2008). Acetylation of H3K18 and also of H4K5 allow association with the BET protein BRD3 (Filippakopoulos and Knapp, 2012). In addition, acetylation at H4K5 also allows the association of BRD4, resulting in chromatin decompaction and induction of transcription (Zhao et al., 2011; Park et al., 2013). This study shows that HIPK2-mediated chromatin decompaction involves the interaction between acetylated lysines and BET domain proteins, as suggested by the inhibitory activity of JQ1 and consistent with an important role of BRD3 and BRD4 for chromatin decompaction and domain boundary formation (Hsu and Blobel, 2017). In this model, the relative acetylation level is shaped by the combined activity of HATs and counteracting HDACs such as HDAC7 to control the kinetics and stoichiometry of histone acetylations. In addition, acetylation levels may also be affected by non-enzymatic functions of HDACs or by the relative levels of the co-substrate acetyl-CoA (Choudhary et al., 2009). In future research it will be important to identify HIPK2-associated chromatin regions by genome-wide studies, as it was successfully achieved for the related kinase DYRK1A (Di Vona et al., 2015). We conducted such ChIP-seq experiments using a highly specific monoclonal HIPK2 antibody, but almost all of the identifed associations with distinct chromatin regions were not specific (data not shown), raising the need to solve this question by alternative approaches in the future.

### HIPK2 as a Co-activator for Estrogen-Induced Gene Expression

This study also uncovered a coactivator function of HIPK2 for estrogen receptor-mediated gene expression, suggesting a more general role of HIP kinases as cofactors for gene expression triggered by nuclear hormone receptors. Early work identified HIPK3 as an activator of androgen receptor function (Moilanen et al., 1998) and a genome-wide RNA interference screen identified HIPK2 as a new regulator of androgen receptor-induced gene expression (Imberg-Kazdan et al., 2013). Also the HIPK-related kinase DYRK1a was found to associate with the androgen receptor-interacting protein 4 (Arip4) to trigger androgen receptor- and glucocorticoid receptor-dependent transcription (Sitz et al., 2004). The coactivator function of HIPK2 for estrogen receptor-mediated gene expression discovered here might also be of pathophysiological relevance, as revealed by the analysis of gene dependency screens for 712 cancer cell lines spanning 3 large-scale RNAi datasets (McFarland et al., 2018). The use of the DEMETER2 computation method to screen for cancers that are specifically vulnerable to loss of HIPK2 revealed breast cancer as the top cancer identities (Supplementary Figure S9). In support to this notion, a direct function of HIPK2 for breast cancer cell migration was described (Nodale et al., 2012) and it will be interesting to find a role of HIPK2 and further members of the HIPK family for hormone-dependent breast cancers in future studies.

## Data Availability Statement

The raw data supporting the conclusions of this article will be made available by the authors, without undue reservation, to any qualified researcher.

## Author Contributions

JH, DB, and SB performed the experiments. MS wrote the first draft of the manuscript. MK corrected and finalized the manuscript. All authors contributed to the article and approved the submitted version.

## Conflict of Interest

The authors declare that the research was conducted in the absence of any commercial or financial relationships that could be construed as a potential conflict of interest.
