# Supplementary material for: Chromatin Targeting of HIPK2 Leads to Acetylation-Dependent Chromatin Decondensation
Source: Front Cell Dev Biol. 2020 Sep 1;8:852. doi: 10.3389/fcell.2020.00852 (PMC7490299; doi:10.3389/fcell.2020.00852)
Supplement: Supplementary file 1 [file Data_Sheet_1.pdf]

Supplementary Data

Chromatin targeting of HIPK2 leads to acetylation-dependent chromatin decondensation

Jana Haas[1], Daniel Bloesel[1], Susanne Bacher[1], Michael Kracht[2] and M. Lienhard SCHMITZ[1,*]

[1]Institute of Biochemistry, Justus-Liebig-University, 35392 Giessen, Germany
Member of the German Center for Lung Research
[2]Rudolf-Buchheim-Institute of Pharmacology, Justus-Liebig-University, 35392 Giessen, Germany
Member of the German Center for Lung Research

Supplementary

S1

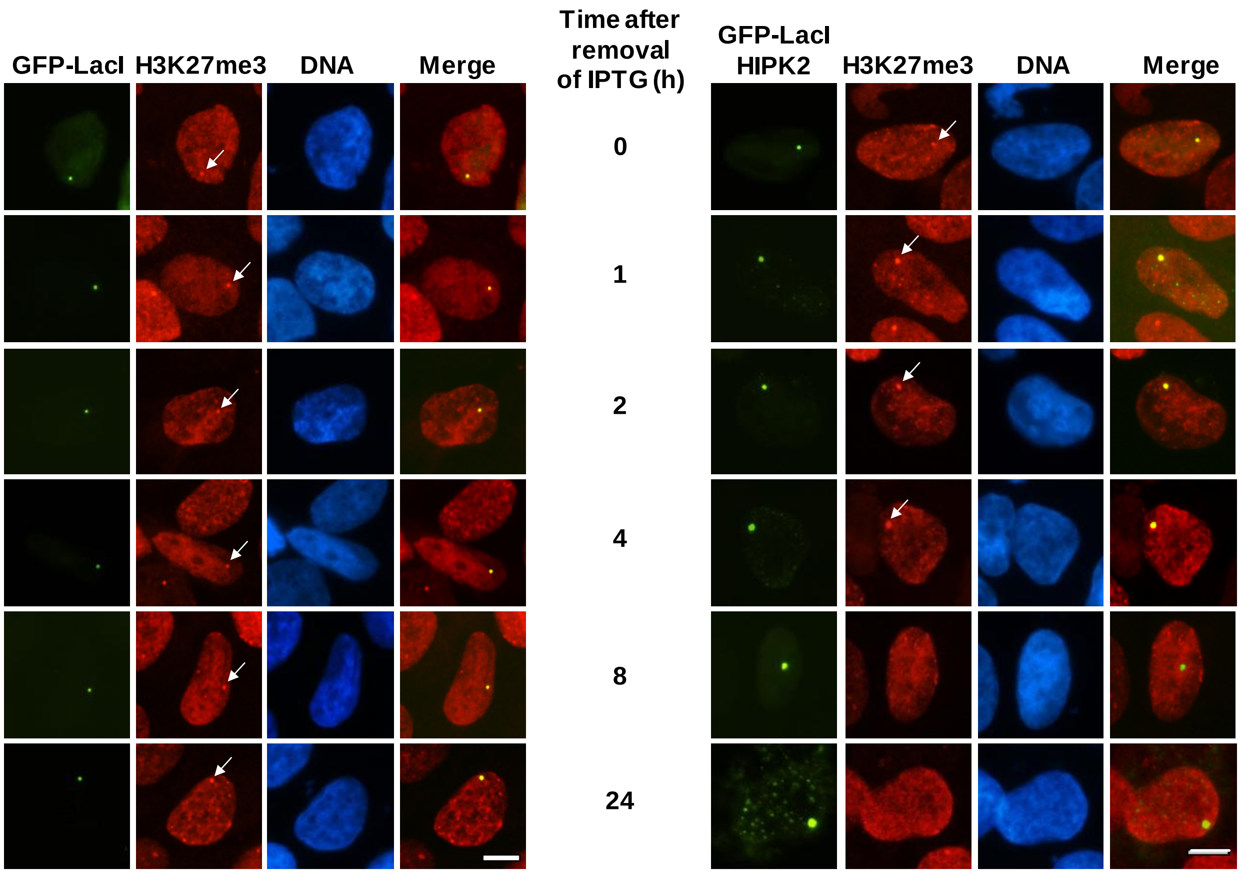

Suppl. Fig. S1. Plasmids encoding GFP-LacI or GFP-LacI-HIPK2 were used to transfect U2OS F42B8 cells. Directly after transfection IPTG (150 µM) was added for 16 h to prevent DNA-binding of LacI. After removal of IPTG cells were further cultivated for the indicated periods and subsequently stained with antibodies detecting H3K27me3. Areas showing co-localization between H3K27me3 spots and lacO arrays are indicated by arrows, bar = 5 µm.

Supplementary

S2

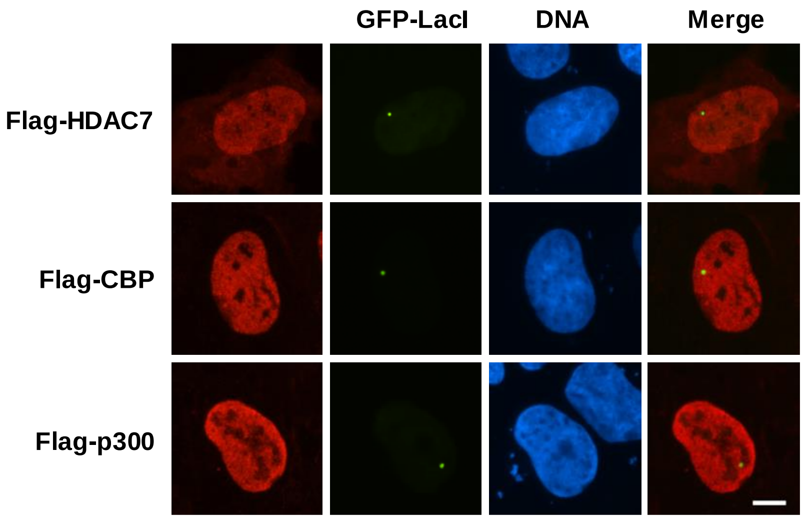

Suppl. Fig. S2. U2OS F42B8 cells were transfected to express moderate amounts of GFP-LacI-HIPK2 together with the indicated epitope-tagged proteins. After one day, cells were fixed and analyzed by immunofluorescence for the intracellular localization of the labelled proteins and the GFP signal, bar = 5 µm.

S3

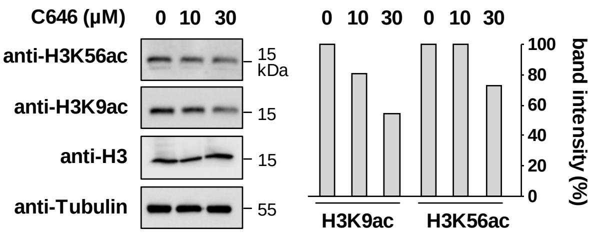

Suppl. Fig. S3. Left: U2OS F42B8 cells were treated for 1 day with the indicated amounts of C646 or vehicle. Cell lysates were prepared and equal amounts of proteins were analyzed by immunoblotting using the indicated antibodies. Right: The protein bands were quantified and normalized to the H3 control. To facilitate comparison the intensities of the vehicle-treated controls were set as 100%.

Supplementary

S4

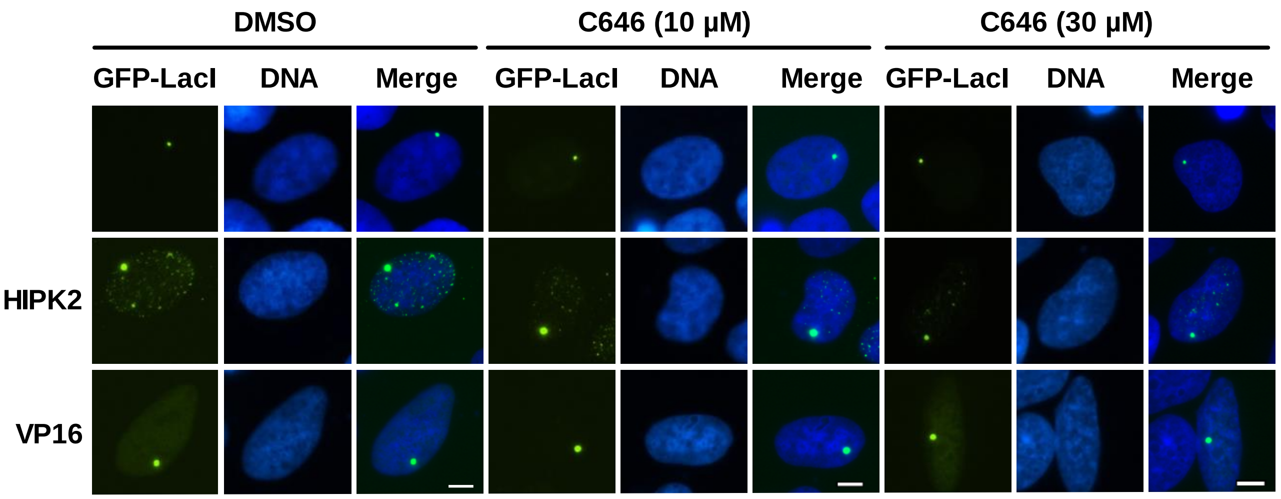

Suppl. Fig. S4. U2OS F42B cells were transfected with the indicated GFP-LacI fusion proteins and incubated with IPTG for 16 h. After removal of IPTG cells were treated with the indicated amounts of C646 or solvent control for one day. Cells were fixed and analyzed by fluorescence microscopy, scale bar = 5 µm.

S5

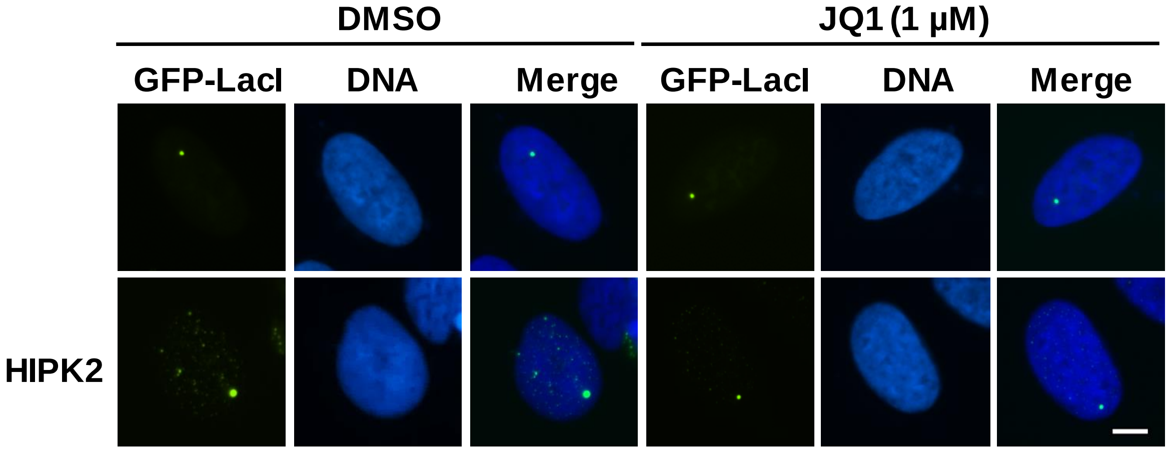

Suppl. Fig. S5. U2OS F42B cells were transfected with the indicated GFP-LacI fusion proteins and incubated with IPTG for 16 h. After removal of IPTG cells were treated with JQ1 or solvent control for one day. Cells were fixed and analyzed by fluorescence microscopy, scale bar = 5 µm.

Supplementary

S6

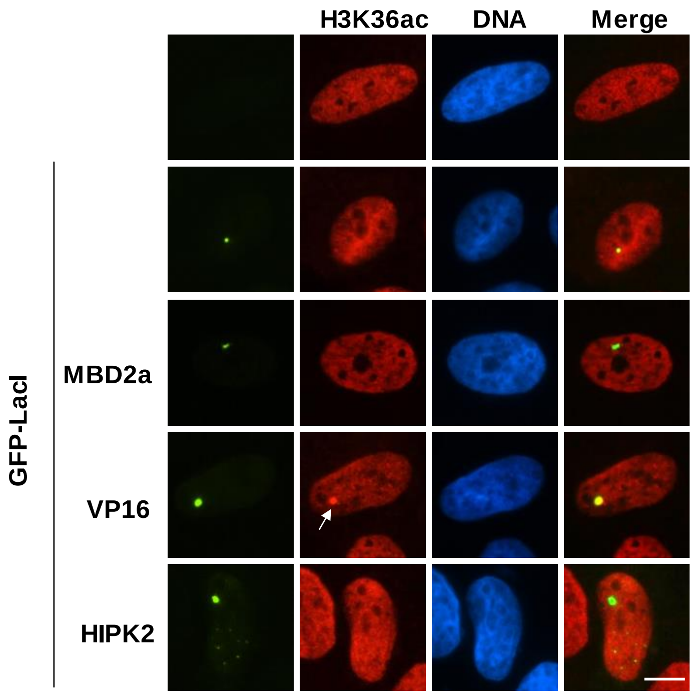

Suppl. Fig. S6. U2OS F42B8 cells were transfected with the indicated plasmids. The next day, cells were fixed and stained for H3K36Ac using specific antibodies. Indirect immunofluorescence was used to analyze co-localization with lacO arrays emitting the GFP signal. An arrows points to a H3K36Ac spot at the lacO repeats, the bar represents 5 µm.

Supplementary

S7

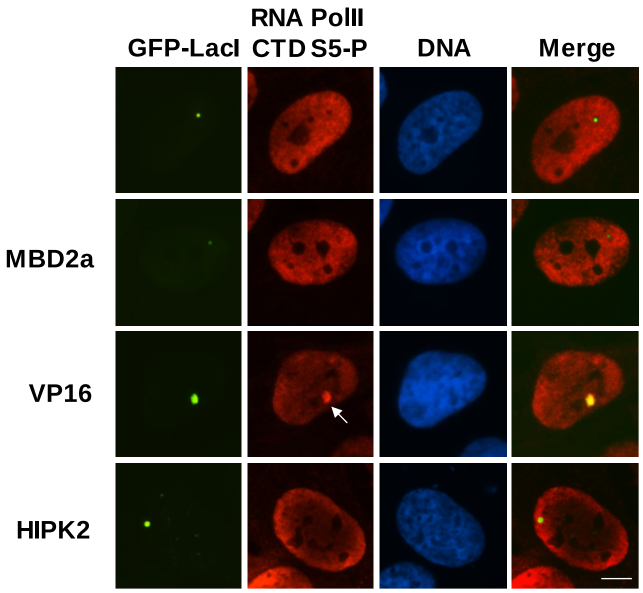

Suppl. Fig. S7. U2OS F42B cells were transfected with the indicated GFP-LacI fusion proteins. The next day, cells were fixed and stained with the indicated phospho-specific antibodies, followed by immunofluorescence analysis. The arrow points to enriched CTD S5 phosphorylation at the region containing the lacO repeats. A representative experiment is shown, bar = 5 µm.

S8

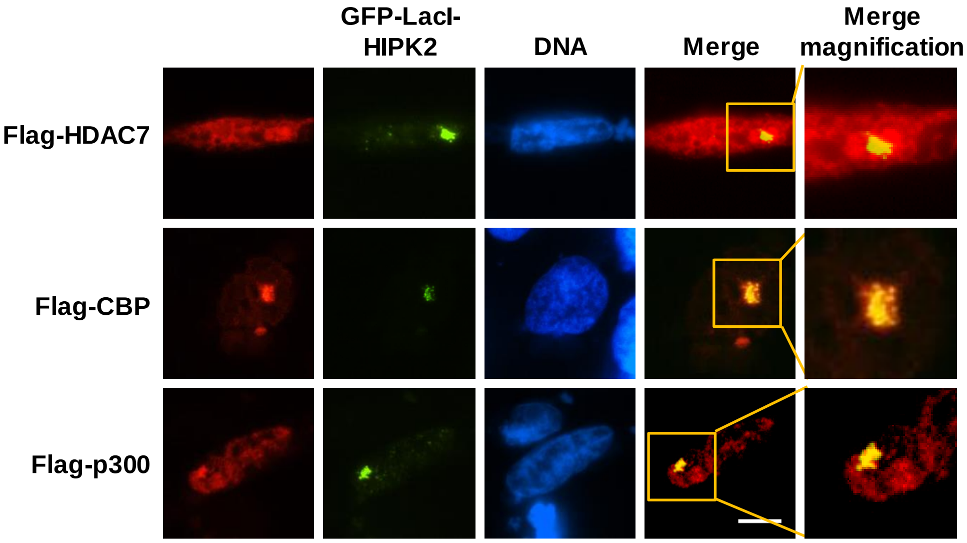

Suppl. Fig. S8. CHO RREB1 cells were transfected to express moderate amounts of GFP-LacI-HIPK2 together with the indicated epitope-tagged proteins. After one day, cells were fixed and analyzed by immunofluorescence for the intracellular localization of the labelled proteins and the GFP signal. The arrows point to lacO arrays, magnifications of the boxed areas are shown at the right. A representative experiment is shown, bar = 5 µm.

Supplementary

S9

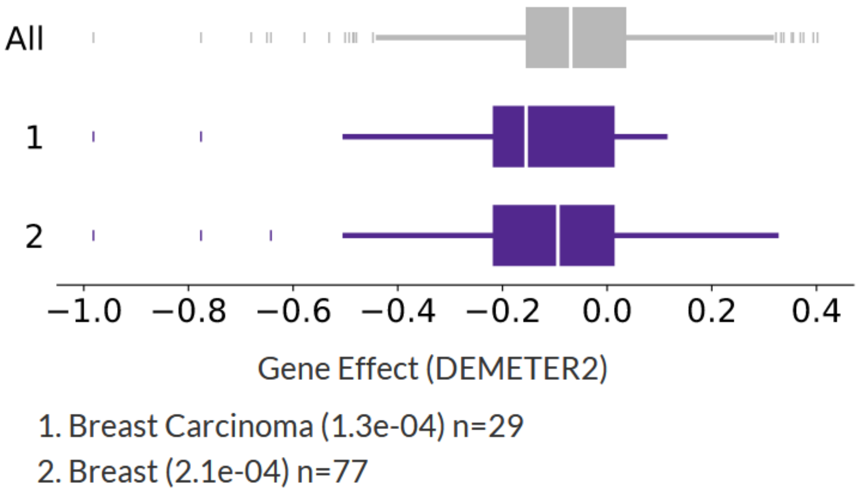

Suppl. Fig. S9. The DEMETER2 algorithm was used (https://depmap.org/R2-D2/) to query for cancer entities vulnerable to HIPK2 depletion.
