# Supplementary material for: Chromatin Targeting of HIPK2 Leads to Acetylation-Dependent Chromatin Decondensation
Source: Front Cell Dev Biol. 2020 Sep 1;8:852. doi: 10.3389/fcell.2020.00852 (PMC7490299; doi:10.3389/fcell.2020.00852)
Supplement: Supplementary file 2 [file Table_1.DOCX]

**Haas et al. supplementary table 1**

**Primary Antibodies**

| **Primary antibody** | **Species** | **Dilution** | **Supplier** |
| --- | --- | --- | --- |
| GFP | mouse mAb  [7.1 und 13.1] | WB: 1:1000 | Roche |
| Tubulin | mouse mAb [E7] | WB: 1:1000 | Sigma |
| Gal4 | rabbit pAb | ChIP: 1:50  WB: 1:1000 | self-made |
| IgG control | rabbit pAb | ChIP: 2 μg | Cell Signaling |
| H3 | rabbit pAb | ChIP: 2 μg  WB: 1:5000 | Abcam |
| H4 | rabbit pAb | ChIP: 2 μg | Millipore |
| H3K27me3 | rabbit pAb | IF: 1:1000 | Diagenode |
| Flag | mouse mAb | WB: 1:5000  IF: 1:1000 | Sigma |
| p300 | rabbit pAb [N-15] | IF: 1:400 | Santa Cruz |
| H3K9ac | rabbit pAb | IF: 1:2000  WB: 1:1000 | Cell Signaling |
| H3K14ac | rabbit pAb | ChIP: 2 μg  IF: 1:500 | Millipore |
| H3K18ac | rabbit pAb | ChIP: 2 μg  IF: 1: 200  WB: 1:1000 | Cell Signaling |
| H3K36ac | rabbit pAb | IF: 1:500 | Diagenode |
| H3K56ac | rabbit pAb | WB: 1:1000 | Cell Signaling |
| H4K5ac | rabbit pAb | ChIP: 2 μg  IF: 1:5000 | Millipore |
| RNA Pol II CTD S2-P | rabbit pAb | IF: 1:500 | Abcam |
| RNA Pol II CTD S5-P | rabbit pAb | IF: 1:500 | Abcam |

**Secondary Antibodies**

| **Secondary antibody** | **conjugated with** | **Dilution** | **Supplier** |
| --- | --- | --- | --- |
| GAR | HRP | WB: 1:5000 | Dianova |
| GAR | Cy3 | IF: 1:3000 | Dianova |
| GAM | HRP | WB: 1:5000 | Dianova |
| GAM | Cy3 | IF: 1:3000 | Dianova |

**Plasmids**

| **Plasmid** | **Origin** | **Reference** |
| --- | --- | --- |
| GFP-LacI | Dr. R. Renkawitz | PMID: 25294833 |
| GFP-LacI-polylinker | Dr. M.L. Schmitz | this study |
| GFP-LacI-MBD2a | Dr. R. Renkawitz | PMID: 23361464 |
| GFP-LacI-VP16 | Dr. C. Rippe | PMID: 29122982 |
| GFP-LacI-HIPK2 | Dr. M.L. Schmitz | this study |
| GFP-LacI-HIPK2 K221A | Dr. M.L. Schmitz | this study |
| GFP-LacI-HIPK2 ΔSIM | Dr. M.L. Schmitz | this study |
| Gal4 | Dr. M.L. Schmitz | PMID: 1935902 |
| Gal4-VP16 | Dr. M.L. Schmitz | PMID: 1935902 |
| Flag-p300 | Dr. M.L. Schmitz | PMID: 22503103 |
| Flag-CBP | Dr. M.L. Schmitz | PMID: 22503103 |
| Flag-HDAC7 | Dr. M.L. Schmitz | PMID: 22503103 |
| lacO-(ERE)3-Luc | Dr. M.L. Schmitz | this study |
| SV40-Renilla Luc | Dr. M.L. Schmitz | PMID: 31242600 |

**DNA-Oligonucleotides**

| **Oligo name** | **purpose** | **Sequence (5´ to 3´)** |
| --- | --- | --- |
| FAIRE ACTB TSS fw | FAIRE | AAAGGCAACTTTCGGAACGG |
| FAIRE ACTB TSS rev | FAIRE | TTCCTCAATCTCGCTCTCGC |
| FAIRE Luci fw | FAIRE | CCCTGGTTCCTGGAACAATTGC |
| FAIRE Luci rev | FAIRE | CCCATATCGTTTCATAGCTTCTGCC |
| ChIP 5xGal4 promotor fw | ChIP | CCAGTGCAAGTGCAGGTG |
| ChIP 5xGal4 promotor rev | ChIP | GCTGGTACCGAGCTCTTAC |
| ChIP gene desert fw | ChIP | AATCACCTTGCATCTGTTTGG |
| ChIP gene desert rev | ChIP | AAAAGGAGAAACCCAGTGGAA |

**Reagents**

| **Reagent** | **Concentration** | **Supplier** |
| --- | --- | --- |
| Hoechst 33342 | working solution 0.1 μg/ml | Abcam |
| (+/-)-JQ1 | 1 μM | Sigma Aldrich |
| C646 | 10 - 30 μM | Sigma Aldrich |
| IPTG | 150 µM | Roth |
| 17β-Estradiol | 10 nM | Sigma Aldrich |
| Kaiser´s Glycerol Gelatin |  | Merck |
| DMEM Gibco (high glucose, no phenol red) |  | Life Technologies |
| Fetal Bovine Serum, charcoal-stripped, one shot Gibco |  | Life Technologies |
| linear PEI |  | PolyScience |
