# Supplementary material for: Chromatin Targeting of HIPK2 Leads to Acetylation-Dependent Chromatin Decondensation
Source: Front Cell Dev Biol. 2020 Sep 1;8:852. doi: 10.3389/fcell.2020.00852 (PMC7490299; doi:10.3389/fcell.2020.00852)
Supplement: Supplementary file 3 [file Table_2.DOCX]

**Supplementary Table 2**

| **Histone modification** | **Immunofluorescence**  **possible** | **Recruitment to** |
| --- | --- | --- |
| H3K36ac | yes | GFP-LacI-VP16 |
| H3K23ac | yes | no |
| H3K18ac | yes | no |
| H3K9ac | yes | no |
| H3K14ac | yes | no |
| H3K27ac | yes | no |
| H4K5ac | yes | no |
| H4K8ac | yes | no |
| H4K12ac | yes | no |
| H4K16ac | yes | no |
| H3K56ac | no | no |
